# Dissecting the multi-scale spatial relationship of earthworm assemblages with soil environmental variability

**DOI:** 10.1186/s12898-014-0026-4

**Published:** 2014-12-05

**Authors:** Juan J Jiménez, Thibaud Decaëns, Patrick Lavelle, Jean-Pierre Rossi

**Affiliations:** Department of Biodiversity Conservation and Ecosystem Restoration, Pyrenean Institute of Ecology (IPE), Spanish National Research Council (CSIC), Jaca (Huesca), ES-22700 Spain; Centre d′Ecologie Fonctionnelle et Evolutive, UMR 5175 CNRS, 1919 Route de Mende, 34293 Montpellier Cedex 5, France; Université Pierre et Marie Curie (Paris 6), 4 place Jussieu, 75005 Paris, France; INRA, UMR CBGP (INRA/IRD/Cirad/Montpellier SupAgro), Campus International de Baillarguet, CS 30016, F-34988 Montferrier-sur-Lez cedex, France

## Abstract

**Background:**

Studying the drivers and determinants of species, population and community spatial patterns is central to ecology. The observed structure of community assemblages is the result of deterministic abiotic (environmental constraints) and biotic factors (positive and negative species interactions), as well as stochastic colonization events (historical contingency). We analyzed the role of multi-scale spatial component of soil environmental variability in structuring earthworm assemblages in a gallery forest from the Colombian “Llanos”. We aimed to disentangle the spatial scales at which species assemblages are structured and determine whether these scales matched those expressed by soil environmental variables. We also tested the hypothesis of the “single tree effect” by exploring the spatial relationships between root-related variables and soil nutrient and physical variables in structuring earthworm assemblages. Multivariate ordination techniques and spatially explicit tools were used, namely cross-correlograms, Principal Coordinates of Neighbor Matrices (PCNM) and variation partitioning analyses.

**Results:**

The relationship between the spatial organization of earthworm assemblages and soil environmental parameters revealed explicitly multi-scale responses. The soil environmental variables that explained nested population structures across the multi-spatial scale gradient differed for earthworms and assemblages at the very-fine- (<10 m) to medium-scale (10–20 m). The root traits were correlated with areas of high soil nutrient contents at a depth of 0–5 cm. Information on the scales of PCNM variables was obtained using variogram modeling. Based on the size of the plot, the PCNM variables were arbitrarily allocated to medium (>30 m), fine (10–20 m) and very fine scales (<10 m). Variation partitioning analysis revealed that the soil environmental variability explained from less than 1% to as much as 48% of the observed earthworm spatial variation.

**Conclusions:**

A large proportion of the spatial variation did not depend on the soil environmental variability for certain species. This finding could indicate the influence of contagious biotic interactions, stochastic factors, or unmeasured relevant soil environmental variables.

**Electronic supplementary material:**

The online version of this article (doi:10.1186/s12898-014-0026-4) contains supplementary material, which is available to authorized users.

## Background

Ecological processes are spatially influenced on various scales, ranging from global to local scales [1,2]. In natural communities, the observed spatial pattern is the result of environmental, biological and/or historical drivers [3], which are not exclusive but rather complementary. The existence of spatial structures of species assemblages suggests the influence of at least one structuring factor: i) a spatially distributed environment is the driving force that structures species assemblages according to niche theory [4]; ii) species are assembled on certain spatial scales through the influence of biotic interactions [5-10]; and iii) historical contingency, according to neutral theory [10,11], or stochastic variations in the history of species arrival [12,13] drive this process, although the scale of the random effect has not been fully identified [14]. It is challenging to determine which process has a larger effect because historical species arrival data and past ecological processes are usually unknown.

When analyzing spatial datasets, striking and puzzling results are found if spatial autocorrelation is ignored because response variables are structured on various spatial scales [15-18]. Specific spatially explicit sampling protocols for targeted organisms and different approaches are needed in soil ecology studies [17,19,20], although these methods must be used with caution [16,21]. Geostatistics [22] allows the assessment of the spatial distribution of soil environmental variability and soil organisms [22,23], but other powerful statistical tools are necessary to model spatial structures on various scales, such as principal coordinates of neighbor matrices (PCNM) [3,24,25]. The PCNM approach is part of the distance-based Moran’s eigenvector map (MEM) analysis, which is included in the spatial eigenfunction family of tools [2,25,26] and is a powerful statistical method to model spatial structures at all scales; in other words, the environmental variability is linked to community structure on a multi-scale level [3,24] to obtain new ecological insights [21]. It has also been used to test and separate the niche from neutral mechanisms that influence the community structure [15], although it may appear over-simplistic [27,28].

To date, few field studies have been performed on the assembly of soil invertebrate communities to infer overall patterns and draw conclusions on the importance of explicitly accounting for multi-spatial scales. Soil organism communities have been reported to be spatially structured due to their response to spatial variability in soil resources [12,19,29-33], allowing the co-existence of competing species within the same patch in spatially heterogeneous environments [32,34]. Although complex spatial patterns have been described for soil invertebrates forming patch assemblages that range from the scale of soil aggregates [35] to those of individual plants [36], agricultural lands and natural ecosystems [37-42], no study has assessed the multi-scale spatial relationship between soil invertebrates and environmental variability thus far. The influence of disturbance and habitat heterogeneity on Carabidae assemblages has been described recently, but only on the landscape scale [19]. Studies and data analysis using these multi-spatial analysis techniques to perform invertebrate community research are needed, even if caution must also be exercised [43]. In this study, we aimed to i) analyze the spatial location of significant patches and gaps of the species assemblages identified, ii) test whether the relationship between species assemblages and soil environmental variability occurs on very fine (<10 m), fine (10–20 m), and medium scales (>30 m), and iii) investigate the spatial relationship between root traits and soil parameters to test the hypothesis of the “single tree effect” [44].

## Results

### Earthworm abundance and soil environmental heterogeneity

A total of 688 earthworms were collected and included seven species (Table 1) with three main ecological categories present [45]: epigeics (litter feeders), *Aymara* sp. and one unclassified species (new genus 1); endogeics (soil feeders), *Andiodrilus* sp., *Andiorrhinus* sp., *Glossodrilus* sp., and one unclassified species (new genus 2); and anecics (soil + litter feeders), *Martiodrilus* sp.

**Table 1:** **Earthworm abundance and main morphological traits**

**Species**	**Family**	**Ecological category** ^**1**^	**Pigmentation**	**Size** ^**2**^ **(mm)**		**Weight** ^**2**^	**N**	**Average density**
				**Length**	**Diam.**	**(g.f.w.)**		**± standard error**
*Andiodrilus* sp.	Glossoscolecidae	Endogeic	Unpigmented	109.0	4.4	1.38	22	3.1 ± 0.7
*Andiorrhinus* sp.	Glossoscolecidae	Endo-anecic	Pink anterodorsal	188.0	7.6	7.10	10	0.1 ± 0.1
*Aymara* sp.	Glossoscolecidae	Epigeic	Dark-red dorsal	58.1	1.5	0.06	15	6.5 ± 1.3
New genus 1	NC^3^	Epigeic	Dark-green dorsal	117.9	3.8	0.69	18	9.5 ± 5.1
*Glossodrilus* sp.	Glossoscolecidae	Endogeic	Unpigmented	83.9	1.5	0.10	13	8.5 ± 1.4
*Martiodrilus* sp.	Glossoscolecidae	Anecic	Dark-grey anterodorsal	194.3	9.3	11.2	29	10.3 ± 1.4
New genus 2	Ocnerodrilidae	Endogeic	Unpigmented	22.8	0.7	0.006	157	24.0 ± 2.6

^1^Epigeic: live and feed on the soil surface; Endogeic: live and feed within the soil; Anecic: live within the soil and dig vertical or semi-vertical burrows to feed on the soil surface (after [45,46]). Endo-anecic worms have characteristics of anecic (anterodorsal pigmentation, flattened rear end) and endogeic worms (horizontal burrowing).

^2^Average biometric data for adults (g.f.w.indicates grams of fresh weight in 4% formalin, gut contents included).

^3^NC: not classified.

**Figure 1:**
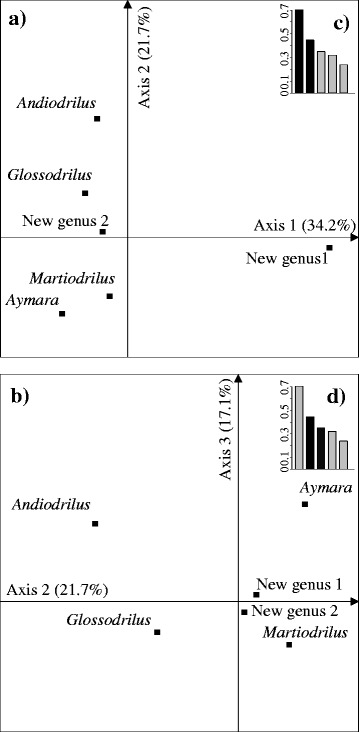
**Ordination plot of species in the factorial plan following correspondence analysis of earthworm density (N m** ^**−2**^ **) in the gallery forest: (a), axes 1 and 2; (b), axes 2 and 3; and (c) and (d), “eigenvalues”.** The species *Andiorrhinus* was not included in the analysis because it only represented 1% of the total earthworm abundance.

The CA extracted three axes (72.9% of the total variance), and these three axes were used to discriminate among the various species assemblages according to the axis selected (Figure 1). Axis I (34.2% of the explained variance) discriminated new genus 1 versus all other species, whereas the second axis (21.7% of the total variance) revealed a clear distinction between endogeic and epigeic + anecic species. Moreover, the position of species along the positive side of axis 2 followed a body size increase among endogeic species. Axis 3 (17.1% of the total variance) separated *Aymara*, *Andiodrilus* and new genus 1 from *Martiodrilus*, *Glossodrilus* and new genus 2.

### Patches and gaps of species assemblages

The SADIE spatial I_a_ index and local v_i_ and v_j_ clustering indices were statistically significant for endogeic species and the group *Martiodrilus*, *Glossodrilus* and new genus 2 (1 anecic +2 endogeics), whereas only the v_j_ index was significant for *Andiodrilus*, *Aymara* and new genus 1, i.e. one endogeic + two epigeics (Table 2). Significant spatial dissociations were found when using those assemblages identified with CA axes, i.e., −0.232 (p = 0.978) between new genus 1 and the rest of species, −0.278 (p = 0.995) between endogeics and epigecis + anecic group, and −0.383 (p = 0.999) between the group *Andiodrilus*, *Aymara* and new genus 1 from *Martiodrilus*, *Glossodrilus* and new genus 2 group.

**Table 2:** **SADIE aggregation indices and associated p levels for the various combinations of earthworm assemblages identified in the three axes extracted from the CA**

**Species assemblages**	**I**_**a**_	**v**_**i**_ **(patch)**	**v**_**j**_ **(gap)**
New genus 1	0.997 NS	0.871 NS	−1.003 NS
Rest of species	1.018 NS	0.944 NS	−1.015 NS
Endogecis	1.414 *	1.485 **	−1.430 *
Epigeics + Anecic	1.011 NS	1.188 NS	−1.061 NS
*Andiodrilus*, *Aymara* and new genus 1	1.222 NS	1.343 *	−1.222 NS
*Martiodrilus, Glossodrilus* and new genus 2	1.453 *	1.320*	−1.428 *

I_a_ = global index of aggregation; v_j_ = mean negative index value (gap); v_i_ = mean positive index value (patch). Departure from randomness is tested using 5,967 permutations. * p < 0.05; ** p < 0.01; NS, not significant.

**Figure 2:**
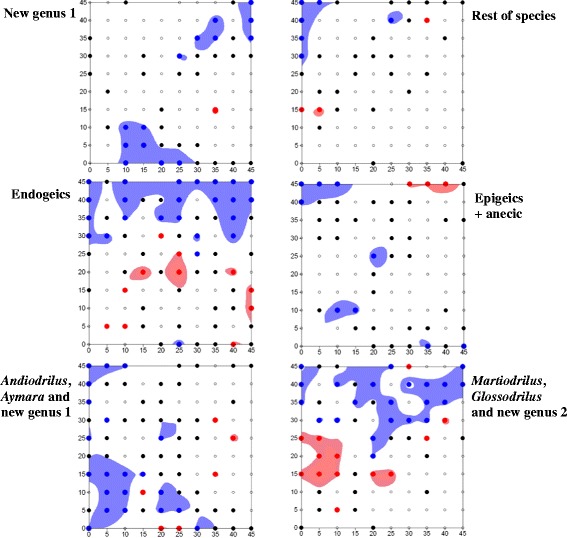
**Overlaid contour and classed post maps (surfer) of SADIE clustering indices for counts of the species assemblages identified in the CA.** Index values > −1.5 represent significant gaps (blue shading and darker blue dots), and index values >1.5 indicate significant patches (red shading and darker red dots). Black dots indicate units for which clustering exceeds expectation, although not significantly (>2 or < −1). Open dots indicate clustering below expectation (<1 or > −1).

**Figure 3:**
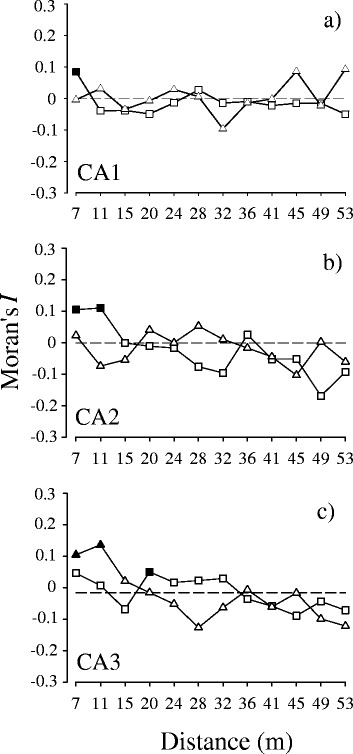
**Correlogram computed using the factorial coordinates for the corresponding positive (□) and negative (Δ) row scores of the three axes extracted from the CA depicting the spatial autocorrelation of a) assemblages CA1+ (New genus 1) and CA1- (rest of species), b) CA2+ (endogeics) and CA2- (epigeics + anecic), and c) CA3+ (** ***Andiodrilus*** **,** ***Avmara*** **, new genus 1) and CA3- (** ***Martiodrilus*** **,** ***Glossodrilus*** **and new genus 2).** Black symbols refer to the lag distances at which the Moran’s I coefficients were significant after progressive Bonferroni corrected p-values (p = 0.05/12; p’ = 0.0042). Only the correlograms of the CA2+ and CA3- assemblages were globally significant.

The number of significant clusters of the earthworm assemblages ranged from 1 (new genus 1) to 9 (endogeics), with gaps occupying a larger area than that of patches (Figure 2). The type of litter and tree root traits may influence the patchy distribution of endogeic earthworms, which is known as the “single tree effect” [44]. The endogeic species assemblage was close to *A. maripa* trees, except for the large patch at the central part of the surveyed plot, where values of the coarse root length and weight (CoRL, CoRW) were the lowest (Figure 3, kriged contour maps).

**Figure 4:**
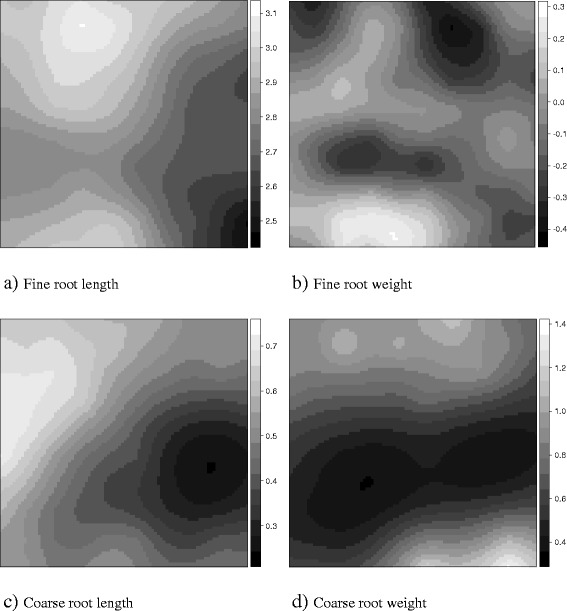
**Kriged maps of root-related variables (log-transformed values): FiRL (a) and CoRL (c), length of fine and coarse roots, respectively (m sample** ^**−1**^ **), and FiRW (b) and CoRW (d), weight of fine and coarse roots, respectively (g dry weight sample** ^**−1**^ **).** Darker areas correspond to lower values.

The identity and location of tree species within the surveyed plot did not appear to explain the observed spatial patterns of the remaining species assemblages:

- ■ new genus 1: a significant gap in the lower half area where multiple tree species, mainly *A. maripa*, were present. The correlogram was not significant (Figure 4a).
- ■ All other species: significant gaps and patches were not linked to areas of tree presence. The correlogram was not significant (Figure 4a).
- ■ Endogeics group: four significant patches close to the location where the *A. maripa* tree species was observed. The correlogram was significant (Figure 4b).
- ■ epigeics + anecic group: same as described for the rest of species (CA1-). The correlogram was not significant (Figure 4b).
- ■ *Andiodrilus, Aymara* and new genus 1: two significant gaps in the lower half area and two significant patches in the plot edge where the *A. maripa* tree species was found. The correlogram was not significant (Figure 4c).
- ■ *Martiodrilus*, *Glossodrilus* and new genus 2 group: one significant patch in the western zone of the plot where trees were present and a large significant gap in the upper part; another two significant patches were in the eastern zone. The correlogram was significant (Figure 4c).

### Cross-correlogram analysis

Regarding the spatial cross-correlation between root-related variables and soil nutrient-related and physical variables, significant positive cross-correlations were identified at short lag distances (h) between the FiRL and SOC_0–5_, P_0–5_ and C:N_5–10_ (Table 3), whereas P_5–10_ showed a significant negative cross-correlation (Monte Carlo permutation). With regard to the CoRL, the cross-correlation at short distances was positive for SOC_5–10_ and C:N_5–10_ and negative for N_5–10_ and P_5–10_. Regarding root biomass, the FiRW showed significant positive spatial cross-correlation with P0-5 and P_5–10_, whereas it was negative for the variables SOC_5–10_, C:N_0–5_ and C:N_5–10_ at short lag distances (Table 3). The CoRW showed a positive spatial relationship with SOC_5–10_ and C:N_5–10_ and a negative relationship with P_5–10_. A significant positive spatial cross-correlation was observed between the FiRL and soil aggregates of less than 5 mm in size, whereas it was negative for larger soil aggregates and moisture at short lag distances (Table 4). Regarding the FiRW, a significant negative spatial cross-correlation at short distances was especially observed for <0.5 and 2–5 mm size soil aggregates, and a positive cross-correlation was observed for >10 mm aggregates and bulk density (BD). Finally, the CoRL showed a positive spatial cross-correlation with 0.125-0.5 and 1–5 mm soil aggregates and hydraulic conductivity, and a negative spatial cross-correlation was observed for >10 mm soil aggregates and soil moisture. The CoRW showed a positive spatial cross-correlation with 0.125-0.25 and 0.5-5 mm size soil aggregates and a negative spatial cross-correlation for >10 mm soil aggregates (Table 4).

**Table 3:** **Cross-correlograms of the root- and nutrient-related soil variables (significant Bonferroni corrected two-sided p-values (0.05/11 = 0.0045) for each distance class were tested using 999 permutations under the null hypothesis)**

**Variables**		**Distance (number of pair points)**											**Global**
Plant below-ground^1^	Nutrient-related	5.0 (360)	8.5 (644)	12.8 (1112)	17.5 (1192)	22.6 (1548)	27.5 (1264)	32.1 (1128)	36.9 (1108)	42.0 (848)	47.3 (516)	52.8 (120)	significance, p’
FiRL	N_0–5_	0.090	0.028	0.021	−0.022	0.026	−0.011	0.005	−0.036	−0.004	−0.069	−0.133	NS
	N_5–10_	−0.015	−0.042	−0.036	−0.034	0.046 *	0.027	0.008	−0.011	0.001	0.033	−0.088	NS
	SOC_0–5_	0.063	0.039	0.027	−0.022	0.012	0.011	0.014	−0.043	−0.009	−0.097 *	−0.076	NS
	SOC_5–10_	0.051	0.077 *	0.082 **	0.039	0.065 **	−0.027	−0.044	−0.092 **	−0.059 *	−0.091 *	−0.128	Significant
	P_0–5_	0.025	0.024	0.060 *	0.016	0.033	0.007	0.010	−0.032	−0.079 *	−0.175 **	−0.063	Significant
	P_5–10_	−0.089 *	−0.133 **	−0.060 *	−0.013	0.025	0.055 *	0.028	0.038	0.001	−0.002	0.044	Significant
	C:N_0–5_	−0.061	0.030	0.010	−0.001	−0.034	0.043	0.025	−0.011	−0.010	−0.045	0.122	NS
	C:N_5–10_	0.053	0.099 *	0.093 **	0.054 *	0.020	−0.043	−0.040	−0.069 **	−0.049	−0.101 **	−0.034	Significant
	Litter	0.081	0.066	−0.020	−0.068 **	−0.008	−0.012	0.035	0.055 *	−0.019	−0.053	−0.022	NS
CoRL	N_0–5_	−0.019	0.028	0.016	0.015	−0.003	0.043	0.015	0.002	−0.057 *	−0.142 **	0.019	Significant
	N_5–10_	−0.146 **	−0.036	−0.013	−0.020	−0.006	0.072 **	0.054 *	0.030	−0.006	−0.085 *	−0.009	NS
	SOC_0–5_	−0.022	0.026	0.012	0.022	0.005	0.041	0.015	−0.005	−0.055	−0.139 **	0.026	NS
	SOC_5–10_	−0.007	0.093 **	0.098 **	0.030	0.001	0.037	−0.003	−0.084 **	−0.098 **	−0.121 **	0.004	Significant
	P_0–5_	0.014	0.048	−0.001	0.028	0.042 *	0.033	0.005	−0.030	−0.079 *	−0.125 **	−0.068	NS
	P_5–10_	−0.127 **	−0.122 **	−0.088 **	−0.028	0.026	0.063 **	0.089 ***	0.076 **	−0.003	−0.042	−0.039	Significant
	C:N_0–5_	−0.012	−0.003	−0.015	0.010	0.021	−0.006	0.003	−0.017	0.007	0.015	0.032	NS
	C:N_5–10_	0.103 *	0.104 *	0.090 ***	0.041	0.005	−0.023	−0.045	−0.094 **	−0.077 *	−0.033	0.007	Significant
	Litter	−0.036	0.005	0.010	−0.014	−0.002	0.012	−0.016	0.031	−0.020	0.035	−0.081	NS
FiRW	N_0–5_	0.028	−0.021	−0.017	−0.062 *	0.012	0.015	0.044	−0.010	0.013	−0.069	0.001	NS
	N_5–10_	0.062	0.010	−0.009	−0.006	0.062 **	−0.013	0.005	−0.047	−0.038	−0.052	−0.061	NS
	SOC_0–5_	−0.043	−0.044	−0.013	−0.059 *	−0.012	0.007	0.058 *	0.014	0.059 *	−0.072	0.044	NS
	SOC_5–10_	−0.075	−0.071 *	−0.036	−0.058 *	0.022	−0.018	−0.021	0.012	0.109 ***	0.108 **	0.125	Significant
	P_0–5_	0.103 *	0.083 *	0.099 ***	0.041	0.022	0.036	0.005	−0.092 **	−0.150 **	−0.243 **	−0.157	Significant
	P_5–10_	0.149 ***	0.145 **	0.094 ***	0.086 **	0.067 **	0.028	−0.074 *	−0.119 **	−0.192 **	−0.213 **	−0.203 *	Significant
	C:N_0–5_	−0.168 **	−0.040	0.011	0.017	−0.053 **	−0.025	0.025	0.051	0.105 **	0.004	0.068	Significant
	C:N_5–10_	−0.111 *	−0.070 *	−0.023	−0.048 *	−0.027	−0.005	−0.020	0.045	0.122 **	0.132 **	0.154	Significant
	Litter	0.060	0.054	−0.061 *	−0.078 **	−0.001	−0.023	0.039	0.020	0.018	0.020	0.192 *	Significant
CoRW	N_0–5_	0.005	0.013	0.027	−0.023	−0.007	−0.003	0.020	0.025	−0.018	−0.084 *	0.015	NS
	N_5–10_	−0.090	−0.020	0.021	−0.049 *	−0.017	0.030	0.069 **	0.037	0.028	−0.110 **	−0.014	NS
	SOC_0–5_	0.003	0.014	0.020	−0.006	0.004	−0.004	0.019	0.004	−0.010	−0.092 *	0.021	NS
	SOC_5–10_	0.025	0.069 *	0.092 ***	−0.018	−0.014	0.015	−0.001	−0.062 *	−0.052	−0.031	−0.025	Significant
	P_0–5_	−0.088	−0.061	−0.060 *	−0.026	0.039	0.048 *	0.051 *	0.058 *	0.018	−0.095 *	−0.148 *	NS
	P_5–10_	−0.183 **	−0.130 **	−0.104 **	−0.045 *	0.018	0.084 **	0.115 ***	0.109 ***	0.024	−0.085 *	−0.103	Significant
	C:N_0–5_	−0.014	0.008	−0.019	0.039	0.029	−0.001	−0.003	−0.052 *	0.017	−0.011	0.037	NS
	C:N_5–10_	0.091 *	0.074 *	0.063 *	0.022	0.003	−0.014	−0.056 *	−0.082 **	−0.064 *	0.062	−0.015	Significant
	Litter	−0.059	0.050	0.011	0.028	−0.017	0.005	−0.033	0.002	0.017	0.003	−0.101	NS

^1^FiRL, fine root length; CoRL, coarse root length; FiRW, fine root weight; CoRW, coarse root weight.

* p < 0.05; ** p < 0.01; *** p < 0.001; NS, not significant.

The number of pair points (within brackets) and the lower and upper limits for each distance class employed while computing the cross-correlograms are indicated.

**Table 4:** **Cross-correlograms of the root- and soil physical variables (significant Bonferroni corrected two-sided p-values (0.05/11 = 0.0045) for each distance class were tested using 999 permutations under the null hypothesis)**

**Variables**		**Distance (number of pair points)**											**Global**
Plant below-ground^1^	Physical	5.0 (360)	8.5 (644)	12.8 (1112)	17.5 (1192)	22.6 (1548)	27.5 (1264)	32.1 (1128)	36.9 (1108)	42.0 (848)	47.3 (516)	52.8 (120)	significance, p’
FiRL	Agg0.053-0.125	−0.121 *	0.010	0.080 **	−0.001	0.036	−0.012	−0.061 *	−0.034	0.046	−0.038	0.067	NS
	Agg0.125-0.25	−0.005	0.103 **	0.106 ***	0.038	0.037	−0.059 *	−0.096 **	−0.055 *	0.030	−0.069	−0.112	Significant
	Agg0.25-0.5	0.010	0.060	0.099 ***	0.049 *	0.043 *	−0.040	−0.068 **	−0.069 **	0.032	−0.131 **	−0.058	Significant
	Agg0.5-1	0.043	0.125 **	0.106 ***	0.054 *	0.029	−0.057	−0.069 *	−0.073 *	−0.008	−0.144 **	−0.098	Significant
	Agg1-2	0.037	0.089 **	0.065 **	0.074 **	0.022	−0.063 **	−0.058 *	−0.037	0.013	−0.133 **	−0.095	Significant
	Agg2-5	0.143 **	0.127 **	0.080 **	0.061 *	0.016	−0.053 *	−0.068 *	−0.073 **	−0.025	−0.096 *	−0.164	Significant
	Agg5-10	−0.038	−0.027	−0.104 **	−0.008	−0.030	0.031	0.023	0.031	0.028	0.025	0.243 **	Significant
	Agg > 10	−0.107 *	−0.127 **	−0.085 **	−0.057 *	−0.006	0.045	0.059 *	0.074 *	−0.005	0.137 **	0.062	Significant
	BD	−0.022	−0.045	−0.025	−0.015	−0.002	0.011	−0.001	0.028	−0.015	0.090 *	0.127	NS
	Comp	−0.128 **	−0.064	−0.047	0.011	0.009	0.003	0.013	0.041	−0.017	0.115 **	0.167 *	NS
	Conduc	0.149 **	0.123 **	0.076 **	−0.002	0.003	−0.044	−0.031	−0.034	−0.029	−0.109 *	−0.211 *	Significant
	Hum	−0.233 **	−0.125 **	−0.118 **	−0.029	0.019	0.048	0.073 *	0.078 **	0.003	0.102 *	0.219 *	Significant
CoRL	Agg0.053-0.125	0.025	0.016	0.040	0.038	0.019	−0.052	−0.022	−0.054	−0.020	0.034	0.030	NS
	Agg0.125-0.25	0.099 *	0.121 **	0.092 ***	0.063 *	0.003	−0.075 **	−0.056 *	−0.094 **	−0.058	−0.006	0.035	Significant
	Agg0.25-0.5	0.087 *	0.111 **	0.093 **	0.074 **	−0.001	−0.049 *	−0.036	−0.108 **	−0.067 *	−0.037	−0.086	Significant
	Agg0.5-1	0.123	0.173 ***	0.095 ***	0.071 **	0.006	−0.063 **	−0.061 *	−0.114 **	−0.078 *	−0.035	−0.052	Significant
	Agg1-2	0.085 *	0.104 **	0.069 *	0.046 *	0.015	−0.038	−0.033	−0.070 *	−0.090 *	−0.023	−0.088	Significant
	Agg2-5	0.156 ***	0.173 ***	0.098 ***	0.058	−0.003	−0.058 *	−0.062 *	−0.102 **	−0.081 *	−0.072	0.008	Significant
	Agg5-10	−0.057	0.000	−0.080 **	−0.051 *	0.007	−0.035	0.029	0.060 *	0.080 **	0.062	0.034	Significant
	Agg > 10	−0.133 **	−0.174 **	−0.082 **	−0.070 **	−0.013	0.067 *	0.059 *	0.119 ***	0.074 *	0.048	0.055	Significant
	BD	−0.061	−0.061	−0.053 *	−0.014	0.008	0.030	0.022	0.013	0.024	0.070 *	−0.068	NS
	Comp	−0.027	−0.064	−0.040	−0.041	−0.006	0.000	0.027	0.035	0.045	0.086 *	0.044	NS
	Conduc	0.068	0.149 ***	0.064 *	0.031	−0.005	−0.015	−0.038	−0.049	−0.070 *	−0.102 **	−0.017	Significant
	Hum	−0.123 **	−0.163 **	−0.114 **	−0.030	0.018	0.011	0.087 ***	0.070 **	0.070 *	0.088 *	0.068	Significant
FiRW	Agg0.053-0.125	−0.099 *	0.017	0.073 **	0.014	0.004	−0.027	−0.050	−0.022	0.055	0.006	0.032	NS
	Agg0.125-0.25	−0.134 **	−0.033	−0.007	−0.033	−0.008	−0.055 *	−0.024	0.041	0.149 ***	0.100 **	0.070	Significant
	Agg0.25-0.5	−0.087 *	−0.043	0.001	−0.041	−0.001	−0.030	−0.010	0.015	0.144 ***	0.016	0.080	Significant
	Agg0.5-1	−0.069	−0.020	0.004	−0.051 *	−0.006	−0.030	0.017	0.013	0.092 ***	−0.008	0.100	Significant
	Agg1-2	−0.073	−0.018	−0.064 *	−0.048	0.001	−0.014	0.032	0.043	0.107 ***	−0.021	0.081	Significant
	Agg2-5	−0.098 *	−0.082 *	−0.080 **	−0.065 **	−0.029	−0.026	0.042	0.057 *	0.174 ***	0.086 *	0.093	Significant
	Agg5-10	0.019	0.048	−0.027	0.034	−0.021	0.041	0.009	−0.040	−0.075 *	0.004	0.055	NS
	Agg > 10	0.091 *	0.056	0.063 **	0.057 *	0.036	0.022	−0.039	−0.047 *	−0.155 **	−0.084 *	−0.087	Significant
	BD	0.090 *	0.112 **	0.049	0.060 *	0.049 *	0.000	−0.098 **	−0.082 **	−0.119 **	0.033	−0.015	Significant
	Comp	−0.072	−0.011	−0.014	0.011	0.016	0.020	−0.044 *	0.028	−0.020	0.096 *	0.006	NS
	Conduc	−0.016	−0.055	−0.042	−0.036	−0.052 *	0.010	0.066 *	0.048 *	0.058 *	−0.023	−0.003	NS
	Hum	−0.037	−0.006	0.002	0.017	0.004	0.029	−0.008	0.009	−0.068 *	0.039	−0.011	NS
CoRW	Agg0.053-0.125	0.056	0.001	0.033	0.038	0.000	−0.069 **	−0.012	−0.042	−0.017	0.079 *	0.044	NS
	Agg0.125-0.25	0.135 **	0.112 **	0.076 **	0.042	−0.020	−0.091 **	−0.050	−0.087 **	−0.042	0.076 *	0.097	Significant
	Agg0.25-0.5	0.103 *	0.081 *	0.073 **	0.040	−0.017	−0.076 **	−0.019	−0.082 **	−0.056	0.047	0.037	NS
	Agg0.5-1	0.120 **	0.113 **	0.070 **	0.049	−0.025	−0.083 **	−0.049	−0.073 *	−0.019	0.041	0.029	Significant
	Agg1-2	0.088	0.103 **	0.052 *	0.042	−0.024	−0.056 *	−0.046	−0.047	−0.055	0.062	0.026	Significant
	Agg2-5	0.169 ***	0.165 ***	0.085 **	0.053 *	−0.042 *	−0.083 **	−0.080 **	−0.076 **	−0.036	0.012	0.122	Significant
	Agg5-10	−0.052	0.038	−0.045	−0.008	−0.017	−0.004	−0.010	0.044	0.080 *	−0.010	−0.062	NS
	Agg > 10	−0.140 **	−0.155 **	−0.081 **	−0.054 *	0.029	0.079 **	0.082 ***	0.090 ***	0.038	−0.034	−0.081	Significant
	BD	−0.106 *	−0.096 *	−0.058 *	0.008	0.009	0.060 *	0.033	0.035	0.001	0.018	−0.109	NS
	Comp	0.003	−0.012	−0.007	−0.018	−0.027	0.026	0.006	0.021	−0.024	0.059	0.063	NS
	Conduc	0.047	0.097 **	0.049 *	0.026	−0.026	−0.029	−0.054 *	−0.024	0.007	−0.054	−0.009	NS
	Hum	−0.102 *	−0.084 *	−0.044	−0.018	0.022	0.019	0.056 *	0.052 *	−0.019	0.023	0.052	NS

^1^FiRL, fine root length; CoRL, coarse root length; FiRW, fine root weight; CoRW, coarse root weight.

* p < 0.05; ** p < 0.01; *** p < 0.001; NS, not significant.

The number of pair points (within brackets) and the lower and upper limits for each distance class employed while computing the cross-correlograms are indicated.

### Decomposing multiple scale spatial patterns of species assemblages

Significant multi-scale spatial structures were obtained for the earthworm community, species and assemblages, especially in the case of new genus 1. The forward selection procedure resulted in various numbers of PCNM variables, ranging from 1 to 9 out of 69 positively autocorrelated spatial eigenvectors (significant Moran’s I at p < 0.05). Variogram modeling [25] provided information on the scales of PCNM variables. The PCNM eigenfunctions selected to model the distribution of earthworm community are depicted in the Additional file 1.

These parameters indicate clear spatial structures on a medium (>30 m), fine (10–20 m) and very fine scale (<10 m), except for *Andiodrilus* (which presented only one significant PCNM). Regarding new genus 1, PCNM3 and PCNM8 defined the medium-scale patterns, whereas PCNM12 and PCNM16 encompassed the fine-scale patterns; PCNM29, PCNM33 and PCNM51 described very fine scales (Additional file 1). The maps of the fitted scores of the significant canonical axes in the PCNM analysis for species (A), species assemblages and the whole community (B) are depicted in Additional file 2.

The significant explanatory environmental variables that best described the multi-spatial structure varied for the earthworm community, species and species assemblages (Table 5). The nutrient-related variables explained much of the structure of new genus 1 on the medium and fine scales, whereas the physical variables were better explained on a very fine scale, such as soil compaction (negatively) and humidity (positively). The variables C_0–5_ (p_corr_ < 0.001) and moisture content (p_corr_ < 0.05) contributed positively to the spatial structure model of new genus 1, whereas C_5–10_ (p_corr_ < 0.05), N_0–5_ (p_corr_ < 0.001), C:N_0–5_ (p < 0.01) and compaction (p_corr_ < 0.05) contributed negatively to medium-scale patterns (Table 5).

**Table 5:** **Significant positive/negative relationship between the spatial characteristics of earthworm species and the soil environmental variables measured in this study**

**Earthworm community, species and assemblages**	**Scales**											
	**Medium >30 m**				**Fine 10-20 m**				**Very fine <10 m**			
	**Vars**	**Coeff**	**P**_**init**_^**§**^	**P**_**corr**_	**Vars**	**Coeff**	**P**_**init**_	**P**_**corr**_	**Vars**	**Coeff**	**P**_**init**_	**P**_**corr**_
Community	P_5–10_	Positive	*	NS	Litter	Negative	*	NS	N_5–10_	Positive	***	**
	-	-	-	-	Comp	Positive	*	NS	C_5–10_	Negative	**	*
	-	-	-	-	-	-	-	-	C:N_5–10_	Positive	**	*
	-	-	-	-	-	-	-	-	Humidity	Negative	*	NS
New genus 1	C_0–5_	Positive	***	***	C_0–5_	Positive	***	***	Comp	Negative	**	*
	N_0–5_	Negative	***	***	N_0–5_	Negative	***	***	Humidity	Positive	**	*
	C:N_0–5_	Negative	***	**	C:N_0–5_	Negative	***	***	-	-	-	-
	Compaction	Negative	**	**	Compaction	Negative	**	**	-	-	-	-
	Humidity	Positive	**	*	C_5–10_	Negative	**	*	-	-	-	-
	C_5–10_	Negative	**	*	Litter	Negative	**	*	-	-	-	-
	-	-	-	-	Humidity	Positive	**	*	-	-	-	-
Andiodrilus	Ag0.25-2	Positive	*	NS	-	-	-	-	-	-	-	-
	Ag2	Positive	*	NS	-	-	-	-	-	-	-	-
	>Ag5	Positive	*	NS	-	-	-	-	-	-	-	-
	<Ag0.25	Positive	*	NS	-	-	-	-	-	-	-	-
Glossodrilus	-	-	-	-	Compaction	Positive	*	NS	-	-	-	-
New genus 2	-	-	-	-	P_0–5_	Positive	**	*	-	-	-	-
	-	-	-	-	Ag0.25.2	Negative	*	NS	-	-	-	-
	-	-	-	-	Ag2	Negative	*	NS	-	-	-	-
	-	-	-	-	>Ag5	Negative	*	NS	-	-	-	-
	-	-	-	-	<Ag0.25	Negative	*	NS	-	-	-	-
	-	-	-	-	Litter	Positive	*	NS	-	-	-	-
	-	-	-	-	N_5–10_	Negative	*	NS	-	-	-	-
	-	-	-	-	PR5	Positive	*	NS	-	-	-	-
Aymara	Compaction	Positive	**	NS	PR20	Negative	*	NS	-	-	-	-
	Humidity	Negative	**	NS	-	-	-	-	-	-	-	-
	FiRL	Positive	*	NS	-	-	-	-	-	-	-	-
Martiodrilus	Humidity	Positive	*	NS	P_5–10_	Positive	*	NS	-	-	-	-
	FiRL	Negative	*	NS								
Endogeics	P_0–5_	Negative	***	**	Litter	Negative	*	NS	-	-	-	-
Epigeics + anecic	Compaction	Negative	**	NS	PR20	Negative	**	NS	PR10	Positive	*	NS
	Litter	Positive	**	NS	BD	Negative	*	NS	-	-	-	-
	Humidity	Positive	*	NS	PR5	Positive	*	NS	-	-	-	-
*Andiodrilus*, *Aymara* and new genus 1	Litter	Positive	**	NS	BD	Negative	**	NS	Compaction	Negative	**	NS
	Compaction	Negative	**	NS	PR20	Negative	*	NS	Humidity	Positive	**	NS
	Humidity	Positive	*	NS	P_5–10_	Positive	*	NS	C_0–5_	Positive	*	NS
	-	-	-	-	N_5–10_	Negative	*	NS	N_0–5_	Negative	*	NS
*Martiodrilus, Glossodrilus* and new genus 2	P_0–5_	Positive	*	NS	-	-	-	-	-	-	-	-
	Compaction	Negative	*	NS	-	-	-	-	-	-	-	-
	CoRW	Negative	*	NS	-	-	-	-	-	-	-	-
	Cond	Negative	*	NS	-	-	-	-	-	-	-	-

^§^A false discovery rate (FDR) procedure was applied to correct the initial p-values (see text for explanation).

* p < 0.05; ** p < 0.01; *** p < 0.001; NS, not significant.

With regard to the endogeic *Andiodrilus* sp., the medium-scale spatial structure was explained by physical variables associated with the size of soil aggregates, although the values were not significant (p_corr_ > 0.05). When species assemblages were used instead, new environmental variables were detected (Table 5), i.e., variable P_0–5_ was negatively correlated to the medium-scale pattern of assemblage of endogeics, and litter contributed negatively to this pattern. For the epigeics and anecic assemblage, litter and moisture contributed positively to the medium-scale spatial structure model, although this contribution was not significant (p_corr_ > 0.05).

### Soil environmental control on earthworm species and assemblage spatial patterns

The variation partitioning analysis revealed differences among species regarding the explanatory variables (Table 6). The entire set of environmental and spatial variables explained the various percentages of variation within the community, species and species assemblage. In the case of the earthworm community, the explained variation was 41.9%, of which 32.3% was explained by the soil environment but not the spatial variables (p = 0.005). The environment and fine-scale structure explained 4.98% of the total variation, whereas the environment and medium-scale structure together explained 2.93%. For the species alone, the R_a_^2^ coefficient for the environmental fraction ranged from 1% for *Aymara* to 48.0% for new genus 1. The medium and fine spatial scales explained 15.4% and 13.4% for *Aymara* and 2% and 2.2% for new genus 1, respectively. The amount of variation explained only by spatial variables independent of the environment differed among species; it ranged from 1.3% to 28.8% for new genus 2 and *Aymara*, respectively (Table 6).

**Table 6:** **Significant PCNM variables (spatial models with eigenfunctions associated with a positive Moran's I) for medium, fine and very fine spatial scales and results of the variation partitioning analysis using adjusted R coefficients (R**_**a**_^**2**^ **), i.e., the amount of variance explained by the environment, the spatial scales and residuals**

**Species and assemblages**	**Number of PCNM eigenvectors**	**Scales**			**Variation partitioning, R**_**a**_^**2**^				**Residual unexplained**
		**Medium**	**Fine**	**Very fine**	**Environment**	**Medium scale**	**Fine, very fine scale**	**Pure spatial**	
Community	6	3, 5, 8	12	33, 51	0.330 **	0.031 NS	0.01 NS	0.018	0.581
*Andiodrilus*	1	-	24	-	0.129 **	0.041 *	-	0.041	0.777
*Aymara*	9	1, 2, 5	15, 20, 24	30, 44, 47	0.002 NS	0.154 **	0.134 **	0.288	0.623
*Glossodrilus*	3	-	13, 24	50	0.053 *	0.081 **	0.056 **	0.141	0.785
*Martiodrilus*	2	5	-	56	0.032 *	0.038 *	0.048 *	0.096	0.867
New genus 1	7	3, 8	12,16	29, 33, 51	0.480 *	0.020 *	0.022 *	0.042	0.369
New genus 2	3	-	-	29, 33, 49	0.176 **	-	0.012 NS	0.013	0.812
Endogeics	4	1, 10	21	65	0.153 **	-	0.015 NS	0.016	0.816
Epigeics + anecic	7	8, 11, 15	20, 33	47, 51	0.145 **	0.098 **	0.118 **	0.235	0.639
*Andiodrilus*, *Aymara* and new genus 1	6	8, 11	20, 33	56, 63	0.198 **	0.123 **	0.077 **	0.222	0.526
*Martiodrilus, Glossodrilus* and new genus 2	2	2, 5			0.101 **	0.058 *	-	0.058	0.762

* p < 0.05; ** p < 0.01; NS, not significant.

## Discussion

Both spatial and environmental variables structured the species, assemblages and earthworm community, although variations were found in the explained contribution of environmental factors, i.e., 33.3% of the total variation of the global spatial structure of the earthworm community was explained by soil environmental variability. The specific soil environmental variables that were significantly linked to particular spatial scales for species and assemblages were also observed in other studies of nematodes in a forest [35]. To a certain extent, our results agree with Hutchinson’s environmental control model [4], although a large portion of the variation was also linked to a purely spatial component (Table 6).

The selected PCNM variables highlighted significant spatial patterns in earthworm assemblages from a gallery forest, allowing us to identify the spatial scale at which the earthworm community was structured. In general, the very fine scale of autocorrelation detected in our study represents spatial patterns of less than 10 m (PCNMs 33, 51; Figure S1 in Additional file 1), fine scales depicted patterns of 15–20 m (PCNMs 12), and medium scales (PCNMs 3, 5, 8) represented spatial patterns of >30 m (see details in Additional file 1). Furthermore, our observation of very fine-, fine- and medium-scale spatial relationships indicates the importance of considering multiple scales during ecological studies of soil organisms. In our study, we carefully chose the scale used to sample earthworms to focus on small-scale patterns. Additional studies are needed to increase the scale of the sampling design, i.e., hundreds of meters to several kilometres.

The influence of environmental constraints on the spatial distribution of species assemblages has previously been demonstrated in a nearby savanna [29]. Moreover, earthworm activity also contributes to soil heterogeneity [33,37]. The joint influence of the soil environment and species-created heterogeneity, i.e., the so-called “functional domain” [47] of soil parameters, could explain the spatial patterns observed on several scales. On very fine scales, the environmental variables associated with the spatial distribution of earthworms were more difficult to detect, i.e., the concentration of soil C_0–5_ and moisture better explained the spatial pattern of new genus 1 on fine and medium scales compared with very fine scales, whereas *Andiodrilus* sp. was mostly associated with physical variables, such as size class aggregated distributions, because this medium-size species produces compact casts that influence the surrounding soil environment [32]. Very fine scales (PCNMs 32 and 50) may be overlooked by classical multivariate analyses, as their relationships may be masked by those of other explanatory variables associated with larger scales, such as PCNM 3. As a detailed analysis of the soil environmental variables was performed within a relatively small area, some of the variation could be attributed to unmeasured variables, leading to incomplete predictions [48]. Moreover, the fraction attributed solely to space was smaller than all other fractions, except for *Aymara* (28.8%), *Glossodrilus* (14.1%), and assemblages epigeics + anecic and *Andiodrilus*, *Aymara* and new genus 1, which represented 23.5% and 22.2% of the total variance, respectively.

The factors affecting the spatial distribution of soil organisms at larger scales include gradients in soil organic matter and vegetation structure [49], whereas at very fine scales (<10 m) earthworm spatial distribution could be influenced by local factors, such as the plant characteristics, soil moisture and micro-topography. A variety of plant species is likely to support important levels of soil heterogeneity [50]. The root architecture or fine-scale spatial patterns within the plant community determine the spatial structures of earthworm populations through fine-scale soil environmental variations, which is known as the “single-tree effect” [44]. Through litter input and root leachates, trees directly influence earthworm populations. They also indirectly influence earthworm populations by altering the soil properties, forming patches beneath tree canopies that influence community structure and ecosystem function [51,52]. Species exclusion was also reported for the savanna grass *Imperata brasiliensis*, as earthworms become injured upon contact with its sharp-pointed roots [38]. In our study, significant positive cross-correlations were found for the CoRL and CoRW and the soil nutrient- and physical-related variables. These close associations between soil variables and vegetation structure have also been described for epigeic invertebrate assemblages [19]. Regarding the importance of soil environmental variables, it should be noted that the influential factors according to our analyses did not operate independently but interacted (e.g., root traits and nutrients); these complex interactions are characteristic of most ecological systems [53].

The idea that species distributions can be linked to key abiotic variables on multiple scales is not new [54]. Our analysis for empirical data has shown that environmental variables are indeed most important on broad scales, whereas purely spatial patterns appear to dominate on finer scales [55]. Applying PCNM analysis toward large-scale assessment of species-environment relationships is a well-established method [19,56,57]. Gilbert and Bennett [43] and Smith and Lundholm [28] criticized the application of variation partitioning to study the relationship between environmental variables and space, although they admitted that it yields useful results when carefully used. Our present analysis supports an optimistic view of this approach. All environmental patterns are spatially correlated on some scale [28], and all ecological processes are spatial to some extent [1]. The common fraction of variance explained jointly by environment and space appears to represent patterns generated by both environmental factors and the limitations on species dispersal [28]. In our study, the highest level of pure spatial variation was obtained for the epigeics + anecic assemblage (23.5%), whereas the endogeics assemblage showed the lowest level (1.6%, Table 6). Species, or even earthworm ecological categories, show specific dispersal behaviors [58], with endogeics typically showing less dispersal than do epigeic and anecic species.

We carefully selected the sampling scale and the spatial statistics tools to address the ecological question at hand [43]. We benefited from our previous knowledge regarding the biology and ecology of the species found in the region where the survey was undertaken [29,32,33,37,38]. Our study lays the groundwork for further detailed analysis of spatial structuring environmental factors and species assemblages on several scales, while also providing clues for developing an accurate and spatially explicit sampling design for earthworm communities. In addition, the utility of the tools used to select species assemblages and analyze their spatial attributes relative to the soil environmental variability was clearly demonstrated. We are confident that our results provide crucial insight into the spatial relationship between species assemblages and soil environmental variability on scales that range several tens of meters. The selection of assemblages from the correspondence analysis, in this case epigeics versus endogeics, and the statistical methods used to draw our conclusions provide insights that improve understanding regarding why particular species assemblages are found at particular sites. From an ecological point of view, our study not only suggests that specific environmental factors determine the structure and spatial distribution of earthworms in the gallery forest but also indicates that a large proportion of unexplained variation exists. Whether this variation is the result of unmeasured soil environmental variables or null model (random) patterns is a subject for further research.

## Conclusions

Earthworms were spatially structured within a relatively small but highly heterogeneous plot; i.e., even at ranges of just a few meters, a multi-scale spatial pattern was observed. The amount of variation jointly explained by the environment and space was not high. However, these sources of variation should not be neglected because they represent unmeasured soil environmental factors and processes that limit species dispersal. Further studies are needed because dispersal traits, for example, remain largely unknown in many earthworm communities. In conclusion, specific abiotic factors were responsible for the observed patterns, and the importance of these patterns needs to be elucidated, even if the multi-scale approach carries additional difficulties and caveats when interpreting the results.

## Methods

### Study area

Sampling was conducted in a gallery forest (GF) at the CORPOICA-CIAT Carimagua field research station in the Eastern Plains of Colombia (4° 37’ N, 71° 19’ W, 170 m a.s.l.). The study area is a well-drained savanna forming a young alluvial plain consisting of Pleistocene and Holocene sediments of Andean origin. The terrain is characterized by open herbaceous savannas where GFs follow a dense braided drainage network of rivers toward the Orinoco catchment. The yearly average temperature and precipitation are 26°C (iso-hyperthermy) and 2,200 mm, respectively, with clayey Oxisols of low pH (4.2-4.4 in water) and fertility, with low available P (1–2 ppm Bray II) and Al saturation >90% (CIAT data).

The plant community of the GF is characterized by several tree species, including *Dendropanax arboreus* (L.) Decne. & Planch. (1854) (Araliaceae), *Enterolobium* spp. (Leguminosae), *Ficus* spp. (Moraceae), *Jacaranda copaia* (Aubl.) (Bignoniaceae), *Copernicia tectorum* (Kunth) Mart. (Caesalpiniaceae), and *Cecropia* sp. (Cecropiaceae). Other species include *Mauritia flexuosa* L.f. and *Mauritiella* (Palmaceae), *Attalea maripa* (Palmaceae), *Nectandra membranacea* (Sw.) Griseb. (Lauraceae), *Didymopanax morototoni* (Aubl.) Decne. & Planch. (Araliaceae), *Virola* sp. (Myristicaceae), and *Hymenaea courbaril* L. (Caesalpiniaceae) [59].

### Earthworms and soil sampling

**Figure 5:**
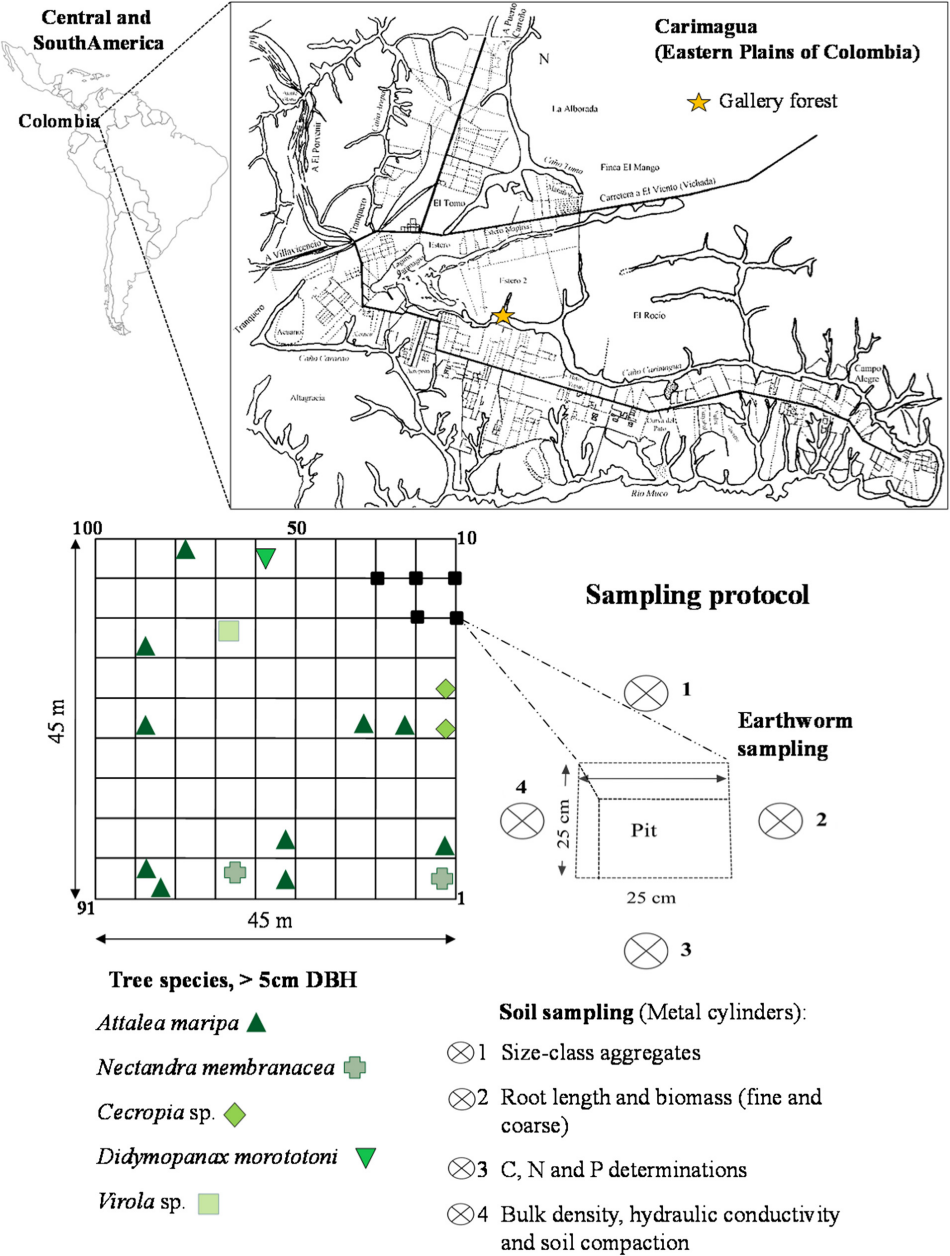
**Sampling protocol used with a regular grid of 10 x 10 sampling points and a 5 m inter-sample distance.** A total of 400 soil samples were collected for the various soil analyses and 100 soil monoliths for earthworm species counts. The location of tree species (>5 m diameter at breast height, DBH) within the surveyed 0.2 ha plot are shown together with the soil pit where the earthworms were sampled, identified and counted, as well as the four soil cores taken for physical and chemical determinations from each of the 100 sampling points.

Earthworms and soil samples were collected at 100 sampling points evenly distributed within a 45 × 45 m^2^ grid with 5 m of inter-sample distance (Figure 5). The earthworms were identified, and their abundance was counted in situ from soil blocks of 25 × 25 cm2 and a depth of 20 cm [60]. Previously, the fresh, tower-like casts deposited on the soil surface by *Martiodrilus* sp. (anecic) were counted at each point within 1 m^2^ quadrats, as they are reliable indicators of the number of active individuals [61].

In total, 400 soil samples were collected for physical and chemical analyses. Soil cores were collected along the four sides of each sampling point (Figure 5). The core method (5 cm depth and 5 cm diam. metal cylinder) was used for bulk density (soil dry mass per volume) following [62]. Water content (soil water per volume and soil water per dry mass) was determined gravimetrically, and hydric conductivity and susceptibility to compaction were also measured [63].

A second core (10 cm depth and 5 cm diam. metal cylinder) was used for soil organic carbon (SOC), nitrogen (N) and phosphorous (P) measurements at 0–5 and 5–10 cm. The soil was then oven dried at 75°C for 48 h and finely ground (<200 μm). A standard colorimetric method was used after digestion in H_2_SO_4_ to measure SOC, and the Kjeldahl method was used to assess the total N. Available P was determined using the Bray-II extraction method. C:N and C:P ratios were calculated as the SOC concentration divided by the total N and P concentrations, respectively.

Another soil core (15 cm depth and 10 cm diam.) was taken to assess the size-class aggregate distribution; ca. 100 g of air-dried soil was mechanically shaken in a sieve column of 4.75, 2, 1, 0.5 and 0.250 mm for 30 min. The last soil core (15 cm depth and 10 cm diam.) was used for root length and biomass quantification. Soil was washed in the lab and sieved to separate the fine (<2 mm) and coarse roots (>2 mm) and then oven dried at 105°C for 48 h.

Resistance to penetration (RP) was measured (3 replicates) using a hand penetrometer at each sampling point. The soil moisture content (volumetric) of the topsoil at the time of sampling was ca. 38% (pF = 2.8).

### Multivariate ordination analysis (CA)

Species abundance (raw data) was analyzed using correspondence analysis (CA). When the species abundance was <5% of the total, it was removed from the species matrix. The extracted factorial axes allowed us to identify various species assemblages, i.e. these were defined according to the sum of the individuals of all species linked to positive or negative row scores of the three axes.

### Species assemblage patches and gaps

SADIE (Spatial Analysis Distance IndicEs) analysis [64,65] was used to assess the presence of significant patches and gaps within species assemblages. The index uses count data, i.e., the total number of individuals who corresponded to any of the assemblages identified per sampling point. A global index of aggregation (I_a_) is computed:

$$ {\mathsf{I}}_{\mathsf{a}}=\mathsf{D}/\mathsf{E}\mathsf{a}, $$

where D is the distance moved to achieve the regular pattern for the observed data and Ea is the arithmetic mean distance to regularity for non-regular randomized samples [64].

I_a_ equals 1 for a random distribution, whereas it is >1 or <1 for either a clumped (aggregated) or regular spatial pattern, respectively [65].

SADIE identifies clusters of high (patches) and low (gaps) mean density, respectively, and these clusters are categorized as v_i_ (positive) and v_j_ (negative cluster index). A patch or gap comprises at least one sample location where the cluster index (v_i_ or v_j_) is significant at the heuristic threshold of 1.5 and −1.5, respectively. Adjacent sample locations with significant index values form a single cluster [65]. The observed v_i_ or v_j_ indices are tested using random permutations against the H_0_ of complete spatial independence of counts [66].

In this study, we used positive and negative row scores extracted from the CA to obtain count data and compute the SADIE v_i_ and v_j_ cluster indices. Factorial coordinates have been used as a typical procedure to analyze the inner structures of data matrices for community analysis [29,33,37-39,67,68]. Because the row scores and factorial coordinates are not count data, which is a requisite for applying SADIE statistics, the various assemblages were obtained by summing the earthworm count data linked to the positive and negative row scores along the CA axes.

Finally, a spatial association/dissociation index was computed between species assemblage pairs [66]. The local association indices calculated from their individual sampling-unit clustering indices are correlated between species assemblage pairs. The observed value of the association index is tested against the H_0_ of complete spatial independence of the counts (based on random permutations). The two-tailed associated probability levels at α = 5% are <0.025 and >0.975 for significant association and dissociation, respectively [66].

### Spatial autocorrelation analysis

In the presence of a spatial dependence, the observation made at one location is more similar to observations made at nearby sites [2], breaking the rule of sample independence for statistical analyses [23]. To assess the degree of spatial autocorrelation, the (semi)-variogram is a function that describes the spatial pattern of any variable with increasing inter-sample (lag) distance. When positive autocorrelation exists, the semi-variance γ(h) increases until it reaches a maximum value (the “sill”) for a given lag distance, which is referred to as the range. This parameter defines the limit of spatial dependence of the variable concerned (detailed in [22,69]). Estimated values of γ(h) are adjusted using a theoretical model [70,71] that is later applied with an interpolation technique called “kriging” to estimate values of the variable under study at non-sampled sites [22]. In our study, interpolated maps were used for root-related variables using only the modeled parameters obtained in the variogram [33].

Cross-variograms can be calculated to assess how two variables co-vary in space [72]. Similar to univariate variograms, cross-covariances may be computed using the values of two distinct variables observed at locations separated by lag h [73]. However, variograms and cross-variograms are not associated with formal testing for departures from randomness (an r^2^ correlation coefficient could be used to adjust the curve). However, in our study, spatial cross-correlation among the root-related variables, soil nutrient contents and physical variables was assessed by calculating the spatial cross-correlogram [55,74]. A spatial autocorrelation coefficient, named Moran’s I, is plotted in the correlogram for increasing distance classes [75]. Data were allocated to 11 distance classes with a minimum of 50 pairs of points for each distance class to compute the cross-correlogram. The significance of the correlogram is tested with a Monte Carlo simulation [20]; it is significant when at least one coefficient is lower than the Bonferroni corrected p′ of α′ = α/k for the k distance classes used [76]. Data normality was tested with a Kolmogorov–Smirnov test; when the normality assumption was not confirmed, a Box–Cox transformation was used [77]. The gstat and ncf packages of the R program 2.15.1 [78] were used to compute the variograms and cross-correlograms and to depict the kriged maps.

### Principal coordinates of neighbor matrices (PCNM) and variation partitioning

The multi-scale spatial analysis of fauna data and soil environmental variability was performed using PCNM analysis [24,79]. This method allows to capture extremely complex structures [80] and is based on the principal coordinate analysis (PCoA) of a truncated pairwise geographic distance matrix between sampling sites [25]. It creates PCNM variables (spatial predictors or eigenfunctions) and a spectral decomposition of spatial relationships from broad to fine spatial scales [81] that is encompassed by the data matrix among sampled sites and then determines to which PCNM variables the response data (uni- or multivariate) respond statistically [79]. Only spatial eigenfunctions associated with positive eigenvalues based on Moran’s I were used to define the spatial structures [3], which represents a highly conservative method due to its penalization of degrees of freedom and adjusted R^2^ statistics [43].

The PCNM variables that significantly contribute toward explaining the species response data are grouped into a small number of submodels, whereas they are normally assigned to broad, intermediate, and fine scales. The predicted values generated for each submodel can then be reanalyzed using canonical analysis against environmental variables to identify the environmental variables associated with species distributions on the scale represented by each submodel [25]. The forward-selection procedure was used [82] to reduce Type I error, as it is known to underestimate the residual variance [83]. In other words, the probability of selecting at least one PCNM is greater than the chosen significance level, even if the response variable is not spatially structured [80]. Appropriate and rigorous approaches for submodel selection have been argued by [43] and [81] is support of improving the methodological developments in MEM-based methods. Scale is generally defined according to the main features of the sampling design, such as the extent of the study area or the size and spacing of the sampling units [84]. Given the dimensions of the plot (45 × 45 m) and our knowledge on the spatial distribution of various earthworm species in the area [33,37,38], the scales were grouped for convenience into medium (>30 m), fine (10–20 m) and very fine (<10 m).

The next step is to relate the spatial components using significant Bonferroni-adjusted p values extracted from the species matrix with soil variables. In other words, species-soil environment regression analysis is performed independently on each scale identified by the PCNM variables. Variogram analysis for each PCNM variable is performed to identify the spatial scale at which the relationship was significant. The lowest value of the Akaike information criterion (AIC) identifies the best spatial model.

Partitioning the variation of a species response data table among two or more explanatory tables is performed using multivariate variation partitioning [85], which determines how much of the species variation is spatially structured and associated with the measured environmental variables [57,80]. The variation partitioning analysis is based on the adjusted R^2^ statistic R_a_^2^ [86], and patterns on finer scales identified by the PCNM variables appear smoother compared to other spatially explicit models, such as nested variograms and filter kriging [79].

The data matrix included count data for 6 earthworm species, 23 soil environmental variables (Additional files 3 and 4) and xy coordinates for 100 sampling points. It has been recommended that fauna data should be detrended and transformed during PCNM analysis. Contrary to correspondence analysis, earthworm abundance data were Hellinger transformed because PCNM has been found to be inappropriate for raw data that includes many null abundances [87]. Earthworm spatial distribution is represented by patches, and clearly meaningful trends are rarely observed; consequently, the data were not detrended. In other words, we were cautious with the use of tests to determine the presence of trends because they are likely not appropriate for patchy patterns. The packages vegan, mass and packfor were used for all the calculations needed during PCNM analysis in R 2.15.1 [78].

### Adjustment of the probability level

The α < 0.05 probability level was corrected using the false discovery rate (FDR) procedure for multiple comparisons [88], in which the power of multiple tests is optimized while controlling for the proportion of significant results that might be Type I errors. The p values from the individual tests are used to perform the corrections and search for significant differences at the corrected probability level. The comparison starts with the highest p value obtained from the individual tests, then each value is checked until encountering the first value that meets the requirement, i.e., the highest p value that is smaller than the corrected p [89]. The transformations include the following:

p(i) ≤ (α/m)*i , where m is the number of tests (variables) and i is the test (variable) ranked in ascending order, i.e., p(1) ≤ ….. ≤ p(m). The final p value corresponded to the following correction:

$$ {p}_{corr} = \left(0.05\kern0.5em \times \kern0.5em  number\  of\kern0.5em  variables\right)/ ranked\ p(maximum) $$

During PCNM analysis, three tests were performed that corresponded to the three spatial scales used: the medium, fine and very fine scales.

## Additional files

Additional file 1
**Plot at the grid nodes of the PCNM variables selected to model earthworm distribution.** A square size is proportional to the value associated to positive (black squares) and negative (white squares) spatial autocorrelation with medium- to fine- and very fine-scale spatial models. Lower order vectors represent broad-scale groupings, and higher order vectors represent more fine-scale groupings. These eigenvectors represent a multi-scale metric for grouping sites, and thus do not represent any computed soil parameter that was measured at sampling sites. The size of the symbols is proportional to the PCNM variables. Additional file 2
**Map of the fitted scores of the significant canonical axes in the PCNM analysis for species (A) and species assemblages and the whole community (B).** The size of squares is proportional to its associated value; black and white colors indicate positive and negative signs of the value associated to the square, respectively. Additional file 3
**Raw count data of species in each sampling point and the resulting assemblages obtained from the positive and negative row scores of the three axes extracted in the correspondence analysis.** These data were later use to calculate SADIE v_i_ and v_j_ cluster indexes. Additional file 4
**Summary statistics of soil environmental variables analysed in this study.**
