# Supplementary material for: Dissecting the multi-scale spatial relationship of earthworm assemblages with soil environmental variability
Source: BMC Ecol. 2014 Dec 5;14:26. doi: 10.1186/s12898-014-0026-4 (PMC4261983; doi:10.1186/s12898-014-0026-4)
Supplement: Additional file 1 — Plot at the grid nodes of the PCNM variables selected to model earthworm distribution. A square size is proportional to the value associated to positive (black squares) and negative (white squares) spatial autocorrelation with medium- to fine- and very fine-scale spatial models. Lower order vectors represent broad-scale groupings, and higher order vectors represent more fine-scale groupings. These eigenvectors represent a multi-scale metric for grouping sites, and thus do not represent any computed soil parameter that was measured at sampling sites. The size of the symbols is proportional to the PCNM variables. [file 12898_2014_26_MOESM1_ESM.docx]

**Additional file 1**

Figure S1. A) Earthworm community.

Figure S1. B) New genus 1

Figure S1. C) *Andiodrilus* sp.

Figure S1. D) *Glossodrilus* sp.

Figure S1. E) New genus 2.

Figure S1. F) *Aymara* sp.

Figure S1. G) *Martiodrilus* sp.

Figure S1. H) Endogeics (CA2+).

Figure S1. I) Epigeics + anecic (CA2-).

Figure S1. J) Epigeics + anecic + New genus 2 (CA3+).

Figure S1. K) *Andiodrilus* + *Glossodrilus* (CA3-).
