# Supplementary material for: Dissecting the multi-scale spatial relationship of earthworm assemblages with soil environmental variability
Source: BMC Ecol. 2014 Dec 5;14:26. doi: 10.1186/s12898-014-0026-4 (PMC4261983; doi:10.1186/s12898-014-0026-4)
Supplement: Additional file 2 — Map of the fitted scores of the significant canonical axes in the PCNM analysis for species (A) and species assemblages and the whole community (B). The size of squares is proportional to its associated value; black and white colors indicate positive and negative signs of the value associated to the square, respectively. [file 12898_2014_26_MOESM2_ESM.docx]

**Additional file 2**

**Species**

|   *Andiodrilus* |   *Aymara* |
| --- | --- |
|   *Glossodrilus* |   *Martiodrilus* |
|   New genus 1 |   New genus 2 |

**Species assemblages + community**

|   Endogeics assemblage |   Epigeics + Anecic assemblage |
| --- | --- |
|   *Andiodrilus*, *Aymara* and new genus 1 assemblage |   *Martiodrilus, Glossodrilus* and new genus 2 assemblage |
|   Community |  |
