# Supplementary material for: Dissecting the multi-scale spatial relationship of earthworm assemblages with soil environmental variability
Source: BMC Ecol. 2014 Dec 5;14:26. doi: 10.1186/s12898-014-0026-4 (PMC4261983; doi:10.1186/s12898-014-0026-4)
Supplement: Additional file 3 — Raw count data of species in each sampling point and the resulting assemblages obtained from the positive and negative row scores of the three axes extracted in the correspondence analysis. These data were later use to calculate SADIE vi and vj cluster indexes. [file 12898_2014_26_MOESM3_ESM.docx]

**Additional file 3**

| Sampling | Coordinates | | Count data of species | | | |  |  |  | Row scores from CA | | | Assemblages in the CA biplot | | | | | | |
| --- | --- | --- | --- | --- | --- | --- | --- | --- | --- | --- | --- | --- | --- | --- | --- | --- | --- | --- | --- |
| point | x | y | Glo | Aym | And | Ng1 | Ng2 | Mrt | Total | Axis 1 | Axis 2 | Axis 3 | CA1+ | CA1- | CA2+ | CA2- | CA3+ | CA3- |  |
| 1 | 0 | 0 | 0 | 0 | 0 | 0 | 45 | 0 | 45 | -0.3773 | 0.0574 | -0.3616 | 0 | 45 | 45 | 0 | 0 | 45 |  |
| 3 | 10 | 0 | 9 | 0 | 9 | 0 | 0 | 0 | 18 | -0.3578 | 2.1038 | 0.6399 | 0 | 18 | 18 | 0 | 18 | 0 |  |
| 5 | 20 | 0 | 0 | 18 | 0 | 0 | 54 | 9 | 81 | -0.4609 | -0.3722 | 0.1823 | 0 | 81 | 0 | 81 | 81 | 0 |  |
| 6 | 25 | 0 | 18 | 36 | 0 | 0 | 36 | 0 | 90 | -0.5469 | -0.3021 | 0.6824 | 0 | 90 | 0 | 90 | 90 | 0 |  |
| 7 | 30 | 0 | 9 | 0 | 9 | 0 | 0 | 0 | 18 | -0.3578 | 2.1038 | 0.6399 | 0 | 18 | 18 | 0 | 18 | 0 |  |
| 8 | 35 | 0 | 0 | 0 | 0 | 0 | 18 | 0 | 18 | -0.3773 | 0.0574 | -0.3616 | 0 | 18 | 18 | 0 | 0 | 18 |  |
| 9 | 40 | 0 | 0 | 0 | 9 | 0 | 54 | 0 | 63 | -0.3627 | 0.4945 | -0.0432 | 0 | 63 | 63 | 0 | 0 | 63 |  |
| 10 | 45 | 0 | 0 | 0 | 9 | 0 | 36 | 9 | 54 | -0.3549 | 0.3947 | -0.0819 | 0 | 54 | 54 | 0 | 0 | 54 |  |
| 11 | 0 | 5 | 0 | 0 | 0 | 0 | 27 | 45 | 72 | -0.3570 | -0.5903 | -0.7058 | 0 | 72 | 0 | 72 | 0 | 72 |  |
| 12 | 5 | 5 | 9 | 0 | 0 | 9 | 27 | 0 | 45 | 0.1501 | 0.2253 | -0.2925 | 45 | 0 | 45 | 0 | 0 | 45 |  |
| 13 | 10 | 5 | 18 | 0 | 9 | 0 | 18 | 0 | 45 | -0.3821 | 1.0826 | -0.0062 | 0 | 45 | 45 | 0 | 0 | 45 |  |
| 14 | 15 | 5 | 9 | 9 | 0 | 0 | 36 | 9 | 63 | -0.4378 | -0.1452 | -0.0836 | 0 | 63 | 0 | 63 | 0 | 63 |  |
| 15 | 20 | 5 | 9 | 9 | 0 | 0 | 18 | 27 | 63 | -0.4284 | -0.4413 | -0.2409 | 0 | 63 | 0 | 63 | 0 | 63 |  |
| 16 | 25 | 5 | 0 | 9 | 0 | 0 | 0 | 0 | 9 | -0.7698 | -1.3579 | 2.3615 | 0 | 9 | 0 | 9 | 9 | 0 |  |
| 17 | 30 | 5 | 18 | 0 | 9 | 0 | 0 | 0 | 27 | -0.3854 | 1.7661 | 0.2307 | 0 | 27 | 27 | 0 | 27 | 0 |  |
| 18 | 35 | 5 | 54 | 0 | 0 | 0 | 9 | 0 | 63 | -0.4314 | 0.9431 | -0.5553 | 0 | 63 | 63 | 0 | 0 | 63 |  |
| 19 | 40 | 5 | 9 | 0 | 18 | 0 | 0 | 0 | 27 | -0.3303 | 2.4415 | 1.0490 | 0 | 27 | 27 | 0 | 27 | 0 |  |
| 20 | 45 | 5 | 27 | 0 | 0 | 0 | 45 | 0 | 72 | -0.4010 | 0.4449 | -0.4464 | 0 | 72 | 72 | 0 | 0 | 72 |  |
| 21 | 0 | 10 | 9 | 9 | 0 | 0 | 18 | 9 | 45 | -0.4619 | -0.2263 | 0.0277 | 0 | 45 | 0 | 45 | 45 | 0 |  |
| 22 | 5 | 10 | 45 | 0 | 9 | 0 | 81 | 27 | 162 | -0.3838 | 0.3417 | -0.3923 | 0 | 162 | 162 | 0 | 0 | 162 |  |
| 23 | 10 | 10 | 0 | 0 | 18 | 0 | 9 | 0 | 27 | -0.3092 | 2.0971 | 1.1244 | 0 | 27 | 27 | 0 | 27 | 0 |  |
| 24 | 15 | 10 | 9 | 9 | 0 | 0 | 18 | 0 | 36 | -0.4912 | -0.0381 | 0.2627 | 0 | 36 | 0 | 36 | 36 | 0 |  |
| 25 | 20 | 10 | 9 | 0 | 0 | 0 | 36 | 9 | 54 | -0.3824 | 0.0569 | -0.4911 | 0 | 54 | 54 | 0 | 0 | 54 |  |
| 26 | 25 | 10 | 9 | 9 | 0 | 9 | 36 | 9 | 72 | -0.0927 | -0.1441 | -0.0468 | 0 | 72 | 0 | 72 | 0 | 72 |  |
| 27 | 30 | 10 | 45 | 0 | 0 | 0 | 9 | 0 | 54 | -0.4299 | 0.9185 | -0.5500 | 0 | 54 | 54 | 0 | 0 | 54 |  |
| 28 | 35 | 10 | 0 | 0 | 9 | 0 | 63 | 27 | 99 | -0.3592 | 0.0530 | -0.3092 | 0 | 99 | 99 | 0 | 0 | 99 |  |
| 29 | 40 | 10 | 0 | 18 | 0 | 0 | 27 | 45 | 90 | -0.4395 | -0.7438 | -0.0923 | 0 | 90 | 0 | 90 | 0 | 90 |  |
| 30 | 45 | 10 | 27 | 9 | 27 | 0 | 9 | 0 | 72 | -0.4117 | 1.4153 | 0.7299 | 0 | 72 | 72 | 0 | 72 | 0 |  |
| 31 | 0 | 15 | 9 | 0 | 0 | 9 | 144 | 0 | 162 | -0.2308 | 0.1041 | -0.3424 | 0 | 162 | 162 | 0 | 0 | 162 |  |
| 32 | 5 | 15 | 0 | 9 | 0 | 0 | 0 | 63 | 72 | -0.3979 | -1.0263 | -0.5031 | 0 | 72 | 0 | 72 | 0 | 72 |  |
| 33 | 10 | 15 | 63 | 0 | 0 | 18 | 72 | 0 | 153 | -0.0856 | 0.4601 | -0.3874 | 0 | 153 | 153 | 0 | 0 | 153 |  |
| 34 | 15 | 15 | 0 | 0 | 0 | 0 | 18 | 18 | 36 | -0.3610 | -0.4607 | -0.6370 | 0 | 36 | 0 | 36 | 0 | 36 |  |
| 35 | 20 | 15 | 0 | 0 | 0 | 0 | 81 | 27 | 108 | -0.3692 | -0.2016 | -0.4993 | 0 | 108 | 0 | 108 | 0 | 108 |  |
| 36 | 25 | 15 | 45 | 0 | 9 | 0 | 18 | 9 | 81 | -0.3974 | 0.8563 | -0.3007 | 0 | 81 | 81 | 0 | 0 | 81 |  |
| 37 | 30 | 15 | 9 | 0 | 0 | 0 | 27 | 0 | 36 | -0.3931 | 0.3158 | -0.4181 | 0 | 36 | 36 | 0 | 0 | 36 |  |
| 38 | 35 | 15 | 0 | 0 | 0 | 171 | 45 | 18 | 234 | 1.5984 | -0.1640 | 0.0138 | 234 | 0 | 0 | 234 | 234 | 0 |  |
| 39 | 40 | 15 | 0 | 0 | 0 | 477 | 0 | 0 | 477 | 2.3229 | -0.1366 | 0.2101 | 477 | 0 | 0 | 477 | 477 | 0 |  |
| 40 | 45 | 15 | 27 | 9 | 0 | 18 | 36 | 0 | 90 | 0.1045 | 0.1871 | -0.0428 | 90 | 0 | 90 | 0 | 0 | 90 |  |
| 41 | 0 | 20 | 0 | 0 | 0 | 9 | 0 | 18 | 27 | 0.5445 | -0.6981 | -0.5382 | 27 | 0 | 0 | 27 | 0 | 27 |  |
| 42 | 5 | 20 | 9 | 0 | 0 | 0 | 36 | 0 | 45 | -0.3900 | 0.2641 | -0.4068 | 45 | 0 | 45 | 0 | 0 | 45 |  |
| 43 | 10 | 20 | 9 | 0 | 0 | 9 | 90 | 9 | 117 | -0.1720 | 0.0423 | -0.3774 | 0 | 117 | 117 | 0 | 0 | 117 |  |
| 44 | 15 | 20 | 0 | 0 | 18 | 36 | 18 | 9 | 81 | 0.8491 | 0.5360 | 0.3266 | 81 | 0 | 81 | 0 | 81 | 0 |  |
| 45 | 20 | 20 | 0 | 0 | 54 | 9 | 27 | 9 | 99 | -0.0732 | 1.6144 | 0.8561 | 0 | 99 | 99 | 0 | 99 | 0 |  |
| 46 | 25 | 20 | 0 | 0 | 0 | 9 | 27 | 0 | 36 | 0.2977 | 0.0089 | -0.2187 | 36 | 0 | 36 | 0 | 0 | 36 |  |
| 47 | 30 | 20 | 0 | 0 | 0 | 0 | 27 | 9 | 36 | -0.3692 | -0.2016 | -0.4993 | 0 | 36 | 0 | 36 | 0 | 36 |  |
| 48 | 35 | 20 | 27 | 0 | 0 | 0 | 0 | 9 | 36 | -0.4165 | 0.5733 | -0.6688 | 0 | 36 | 36 | 0 | 0 | 36 |  |
| 49 | 40 | 20 | 9 | 0 | 0 | 0 | 72 | 9 | 90 | -0.3804 | 0.0571 | -0.4393 | 0 | 90 | 90 | 0 | 0 | 90 |  |
| 50 | 45 | 20 | 9 | 0 | 9 | 0 | 0 | 0 | 18 | -0.3578 | 2.1038 | 0.6399 | 0 | 18 | 18 | 0 | 18 | 0 |  |
| 51 | 0 | 25 | 0 | 0 | 0 | 0 | 27 | 18 | 45 | -0.3643 | -0.3571 | -0.5819 | 0 | 45 | 0 | 45 | 0 | 45 |  |
| 52 | 5 | 25 | 9 | 0 | 0 | 0 | 0 | 63 | 72 | -0.3567 | -0.7202 | -0.8717 | 0 | 72 | 0 | 72 | 0 | 72 |  |
| 53 | 10 | 25 | 0 | 9 | 0 | 0 | 27 | 27 | 63 | -0.4194 | -0.5889 | -0.2086 | 0 | 63 | 0 | 63 | 0 | 63 |  |
| 54 | 15 | 25 | 18 | 0 | 0 | 18 | 27 | 27 | 90 | 0.1599 | -0.0856 | -0.4577 | 90 | 0 | 0 | 90 | 0 | 90 |  |
| 55 | 20 | 25 | 0 | 0 | 0 | 0 | 0 | 27 | 27 | -0.3447 | -0.9789 | -0.9123 | 0 | 27 | 0 | 27 | 0 | 27 |  |
| 56 | 25 | 25 | 9 | 0 | 0 | 0 | 27 | 0 | 36 | -0.3931 | 0.3158 | -0.4181 | 0 | 36 | 36 | 0 | 0 | 36 |  |
| 57 | 30 | 25 | 0 | 0 | 0 | 0 | 27 | 36 | 63 | -0.3587 | -0.5347 | -0.6763 | 0 | 63 | 0 | 63 | 0 | 63 |  |
| 58 | 35 | 25 | 36 | 0 | 0 | 0 | 45 | 9 | 90 | -0.3993 | 0.3671 | -0.5071 | 0 | 90 | 90 | 0 | 0 | 90 |  |
| 59 | 40 | 25 | 0 | 9 | 0 | 0 | 27 | 0 | 36 | -0.4754 | -0.2964 | 0.3192 | 0 | 36 | 0 | 36 | 36 | 0 |  |
| 60 | 45 | 25 | 18 | 9 | 18 | 0 | 99 | 0 | 144 | -0.3970 | 0.4806 | 0.0590 | 0 | 144 | 144 | 0 | 144 | 0 |  |
| 61 | 0 | 30 | 0 | 9 | 0 | 0 | 9 | 27 | 45 | -0.4363 | -0.8474 | -0.1474 | 0 | 45 | 0 | 45 | 0 | 45 |  |
| 62 | 5 | 30 | 0 | 9 | 0 | 0 | 0 | 0 | 9 | -0.7698 | -1.3579 | 2.3615 | 0 | 9 | 0 | 9 | 9 | 0 |  |
| 63 | 10 | 30 | 0 | 0 | 0 | 18 | 0 | 0 | 18 | 2.3229 | -0.1366 | 0.2101 | 18 | 0 | 0 | 18 | 18 | 0 |  |
| 64 | 15 | 30 | 18 | 0 | 18 | 36 | 36 | 0 | 108 | 0.5293 | 0.6749 | 0.1628 | 108 | 0 | 108 | 0 | 108 | 0 |  |
| 65 | 20 | 30 | 0 | 0 | 0 | 9 | 27 | 0 | 36 | 0.2977 | 0.0089 | -0.2187 | 36 | 0 | 36 | 0 | 0 | 36 |  |
| 66 | 25 | 30 | 0 | 0 | 9 | 0 | 0 | 0 | 9 | -0.2752 | 3.1170 | 1.8674 | 0 | 9 | 9 | 0 | 9 | 0 |  |
| 67 | 30 | 30 | 0 | 18 | 0 | 0 | 18 | 0 | 36 | -0.5735 | -0.6502 | 0.9999 | 0 | 36 | 0 | 36 | 36 | 0 |  |
| 68 | 35 | 30 | 0 | 9 | 0 | 0 | 9 | 18 | 36 | -0.4591 | -0.8146 | 0.0438 | 0 | 36 | 0 | 36 | 36 | 0 |  |
| 69 | 40 | 30 | 0 | 0 | 0 | 9 | 0 | 45 | 54 | 0.0999 | -0.8385 | -0.7253 | 54 | 0 | 0 | 54 | 0 | 54 |  |
| 70 | 45 | 30 | 9 | 0 | 0 | 0 | 18 | 36 | 63 | -0.3677 | -0.3871 | -0.7086 | 0 | 63 | 0 | 63 | 0 | 63 |  |
| 71 | 0 | 35 | 0 | 9 | 9 | 0 | 9 | 0 | 27 | -0.4741 | 0.6055 | 1.2891 | 0 | 27 | 27 | 0 | 27 | 0 |  |
| 72 | 5 | 35 | 45 | 0 | 0 | 0 | 27 | 0 | 72 | -0.4168 | 0.7032 | -0.5029 | 0 | 72 | 72 | 0 | 0 | 72 |  |
| 73 | 10 | 35 | 0 | 0 | 0 | 36 | 18 | 9 | 63 | 1.1703 | -0.2015 | -0.1136 | 63 | 0 | 0 | 63 | 0 | 63 |  |
| 74 | 15 | 35 | 9 | 90 | 0 | 0 | 63 | 0 | 162 | -0.5989 | -0.6715 | 1.1387 | 0 | 162 | 0 | 162 | 162 | 0 |  |
| 75 | 20 | 35 | 18 | 54 | 0 | 0 | 54 | 18 | 144 | -0.5283 | -0.4737 | 0.5625 | 0 | 144 | 0 | 144 | 144 | 0 |  |
| 76 | 25 | 35 | 0 | 36 | 0 | 0 | 0 | 0 | 36 | -0.7698 | -1.3579 | 2.3615 | 0 | 36 | 0 | 36 | 36 | 0 |  |
| 77 | 30 | 35 | 9 | 0 | 0 | 0 | 36 | 9 | 54 | -0.3824 | 0.0569 | -0.4911 | 0 | 54 | 54 | 0 | 0 | 54 |  |
| 78 | 35 | 35 | 0 | 9 | 0 | 0 | 0 | 9 | 18 | -0.5572 | -1.1684 | 0.7246 | 0 | 18 | 0 | 18 | 18 | 0 |  |
| 79 | 40 | 35 | 45 | 36 | 0 | 0 | 72 | 27 | 180 | -0.4667 | -0.1228 | 0.0439 | 0 | 180 | 0 | 180 | 180 | 0 |  |
| 80 | 45 | 35 | 0 | 9 | 0 | 0 | 18 | 0 | 27 | -0.5081 | -0.4143 | 0.5461 | 0 | 27 | 0 | 27 | 27 | 0 |  |
| 81 | 0 | 40 | 9 | 0 | 0 | 0 | 9 | 9 | 27 | -0.3875 | 0.0564 | -0.6205 | 0 | 27 | 27 | 0 | 0 | 27 |  |
| 82 | 5 | 40 | 0 | 0 | 0 | 0 | 18 | 0 | 18 | -0.3773 | 0.0574 | -0.3616 | 0 | 18 | 18 | 0 | 0 | 18 |  |
| 83 | 10 | 40 | 0 | 9 | 0 | 0 | 9 | 0 | 18 | -0.5735 | -0.6502 | 0.9999 | 0 | 18 | 0 | 18 | 18 | 0 |  |
| 84 | 15 | 40 | 0 | 9 | 0 | 0 | 0 | 0 | 9 | -0.7698 | -1.3579 | 2.3615 | 0 | 9 | 0 | 18 | 18 | 0 |  |
| 85 | 20 | 40 | 0 | 27 | 9 | 0 | 0 | 9 | 45 | -0.5858 | -0.3871 | 1.6079 | 0 | 45 | 0 | 45 | 45 | 0 |  |
| 86 | 25 | 40 | 0 | 0 | 0 | 27 | 9 | 18 | 54 | 0.9837 | -0.3850 | -0.2593 | 54 | 0 | 0 | 54 | 0 | 54 |  |
| 87 | 30 | 40 | 9 | 0 | 0 | 0 | 9 | 0 | 18 | -0.4089 | 0.5741 | -0.4746 | 0 | 18 | 18 | 0 | 0 | 18 |  |
| 88 | 35 | 40 | 0 | 18 | 0 | 0 | 18 | 36 | 72 | -0.4591 | -0.8146 | 0.0438 | 0 | 72 | 0 | 72 | 72 | 0 |  |
| 89 | 40 | 40 | 0 | 0 | 18 | 0 | 0 | 0 | 18 | -0.2752 | 3.1170 | 1.8674 | 0 | 18 | 18 | 0 | 18 | 0 |  |
| 90 | 45 | 40 | 0 | 0 | 0 | 0 | 27 | 9 | 36 | -0.3692 | -0.2016 | -0.4993 | 0 | 36 | 0 | 36 | 0 | 36 |  |
| 91 | 0 | 45 | 0 | 18 | 0 | 0 | 0 | 0 | 18 | -0.7698 | -1.3579 | 2.3615 | 0 | 18 | 0 | 18 | 18 | 0 |  |
| 92 | 5 | 45 | 9 | 0 | 0 | 9 | 0 | 9 | 27 | 0.5126 | -0.0083 | -0.4300 | 27 | 0 | 0 | 27 | 0 | 27 |  |
| 93 | 10 | 45 | 0 | 0 | 0 | 0 | 27 | 0 | 27 | -0.3773 | 0.0574 | -0.3616 | 0 | 27 | 27 | 0 | 0 | 27 |  |
| 96 | 25 | 45 | 0 | 9 | 0 | 0 | 0 | 0 | 9 | -0.7698 | -1.3579 | 2.3615 | 0 | 9 | 0 | 9 | 9 | 0 |  |
| 97 | 30 | 45 | 18 | 0 | 0 | 9 | 0 | 27 | 54 | 0.0680 | -0.1487 | -0.6170 | 54 | 0 | 0 | 54 | 0 | 54 |  |
| 98 | 35 | 45 | 0 | 9 | 0 | 0 | 36 | 27 | 72 | -0.4142 | -0.5081 | -0.2277 | 0 | 72 | 0 | 72 | 0 | 72 |  |
| 99 | 40 | 45 | 0 | 45 | 0 | 0 | 18 | 9 | 72 | -0.6185 | -0.9567 | 1.2715 | 0 | 72 | 0 | 72 | 72 | 0 |  |
| 100 | 45 | 45 | 0 | 36 | 0 | 0 | 36 | 36 | 108 | -0.4973 | -0.7598 | 0.3625 | 0 | 108 | 0 | 108 | 108 | 0 |  |

^1^ Points 2, 4, 94 and 95 were not included in the analysis as these rows contained only zero values and were not allowed in the correspondence analysis.

^2^ Glo = Glossodrilus sp., Aym = Aymara sp., And = Andiodrilus sp., Ng1 = new genus 1, Ng2 = new genus 2, Mrt = Martiodrilus sp. Andiorrhinus sp. was not included in the analysis as it only was found in one sample (<5%).
