# Supplementary material for: Dissecting the multi-scale spatial relationship of earthworm assemblages with soil environmental variability
Source: BMC Ecol. 2014 Dec 5;14:26. doi: 10.1186/s12898-014-0026-4 (PMC4261983; doi:10.1186/s12898-014-0026-4)
Supplement: Additional file 4 — Summary statistics of soil environmental variables analysed in this study. [file 12898_2014_26_MOESM4_ESM.docx]

**Additional file 4**

| Variables^1^ | Mean | SD | C.V. | Max. | Min. | Median | Kurtosis | Skewness |
| --- | --- | --- | --- | --- | --- | --- | --- | --- |
| Litter (g m^-2^) | 29.75 | 18.64 | 0.63 | 102.64 | 4.23 | 25.37 | 2.64 | 1.48 |
| Moisture (w/w %) | 37.76 | 3.35 | 0.09 | 45.51 | 29.79 | 37.93 | -0.50 | -0.11 |
| P_0-5_ (ppm) | 498.72 | 55.86 | 0.11 | 688.06 | 380.17 | 495.86 | 0.56 | 0.43 |
| P_5-10_ (ppm) | 456.80 | 92.99 | 0.20 | 974.74 | 135.89 | 458.06 | 9.56 | 1.31 |
| SOC_0-5_ (g kg^-1^) | 37.42 | 7.30 | 0.20 | 74.98 | 22.44 | 37.49 | 6.15 | 1.38 |
| SOC_5-10_ (g kg^-1^) | 23.73 | 4.28 | 0.18 | 46.99 | 11.35 | 23.63 | 8.80 | 1.44 |
| N_0-5_ (g kg^-1^) | 2.48 | 0.47 | 0.19 | 4.39 | 1.22 | 2.47 | 1.95 | 0.60 |
| N_5-10_ (g kg^-1^) | 1.67 | 0.25 | 0.15 | 2.60 | 0.67 | 1.67 | 2.99 | 0.03 |
| C:N_0-5_ | 15.16 | 1.24 | 0.08 | 18.52 | 11.79 | 15.00 | 0.52 | 0.24 |
| C:N_5-10_ | 14.52 | 3.62 | 0.25 | 42.03 | 4.37 | 14.36 | 34.69 | 4.55 |
| FiRL (m sample^-1^)^†^ | 18.52 | 8.0 | 0.43 | 48.80 | 4.20 | 17.60 | 2.18 | 1.22 |
| CoRL (m sample^-1^) | 0.49 | 0.54 | 1.10 | 2.50 | 0 | 0.40 | 0.80 | 1.04 |
| FiRW (g sample^-1^) | 1.0 | 0.43 | 0.43 | 2.73 | 0.26 | 0.92 | 2.58 | 1.19 |
| CoRW (g sample^-1^) | 0.69 | 1.27 | 1.84 | 6.33 | 0 | 0.25 | 8.75 | 2.90 |
| PR2 (MPa) | 3.07 | 3.20 | 1.04 | 9.00 | 0 | 0.75 | -1.55 | 0.46 |
| PR5 (MPa) | 3.46 | 3.59 | 1.04 | 10.50 | 0 | 0.92 | -1.58 | 0.42 |
| PR10 (MPa) | 3.76 | 3.98 | 1.06 | 12.00 | 0 | 0.83 | -1.46 | 0.46 |
| <0.250 Agg (%) | 4.72 | 1.15 | 0.24 | 8.02 | 2.21 | 4.72 | -0.04 | 0.16 |
| Agg0.250-1 (%) | 11.32 | 2.27 | 0.20 | 16.96 | 4.51 | 11.54 | 0.36 | -0.23 |
| Agg1-5 (%) | 37.32 | 6.76 | 0.18 | 51.35 | 18.95 | 37.62 | -0.44 | -0.13 |
| Agg>5 (%) | 46.64 | 9.38 | 0.20 | 74.03 | 24.85 | 46.40 | 0.08 | 0.23 |
| BD (Kg m^-3^) | 1.24 | 0.10 | 0.08 | 1.43 | 0.77 | 1.24 | 6.12 | -1.51 |
| Comp (%) | 80.41 | 6.30 | 0.08 | 91.00 | 55.20 | 81.90 | 1.52 | -0.91 |
| Cond (cm h^-1^) | 42.10 | 48.48 | 1.15 | 196.55 | 0.56 | 19.64 | 1.64 | 1.56 |

^1^ P, Phosphorous; SOC, Soil organic Carbon; N, Nitrogen; FiRL, Fine root length; CoRL, Coarse root length; FiRW, Fine root weight; CoRW, Coarse root weight; PR, Penetration resistance; <0.250 Agg, Aggregates <0.250 mm; BD, Bulk density; Comp, Compaction (Susceptibility to); Cond, Hydraulic conductivity. 0-5: soil depth 0- 5 cm; 5-10: soil depth 5-10 cm; MPa: MegaPascals.

^†^ Sample refers to a soil core of 10 cm dia. and 15 cm long (1,178.1 cm^3^)
